# The Extended Clinical Phenotype of 26 Patients with Chronic Mucocutaneous Candidiasis due to Gain-of-Function Mutations in STAT1

**DOI:** 10.1007/s10875-015-0214-9

**Published:** 2015-11-25

**Authors:** Mark Depner, Sebastian Fuchs, Jan Raabe, Natalie Frede, Cristina Glocker, Rainer Doffinger, Effrossyni Gkrania-Klotsas, Dinakantha Kumararatne, T. Prescott Atkinson, Harry W. Schroeder, Tim Niehues, Gregor Dückers, Asbjørg Stray-Pedersen, Ulrich Baumann, Reinhold Schmidt, Jose L. Franco, Julio Orrego, Moshe Ben-Shoshan, Christine McCusker, Cristina Miuki Abe Jacob, Magda Carneiro-Sampaio, Lisa A. Devlin, J. David M. Edgar, Paul Henderson, Richard K. Russell, Anne-Bine Skytte, Suranjith L. Seneviratne, Jennifer Wanders, Hans Stauss, Isabelle Meyts, Leen Moens, Milos Jesenak, Robin Kobbe, Stephan Borte, Michael Borte, Dowain A. Wright, David Hagin, Troy R. Torgerson, Bodo Grimbacher

**Affiliations:** Center for Chronic Immunodeficiency, University Medical Center Freiburg, Engesser Straße 4, 79108 Freiburg, Germany; Faculty of Biology, University of Freiburg, Freiburg, Germany; Addenbrooke’s Hospital, Cambridge, UK; University of Alabama at Birmingham, Birmingham, USA; Helios Kliniken, Childrens Hospital, Krefeld, Germany; Department of Medical Genetics, Oslo University Hospital, Oslo, Norway; Medical University of Hannover, Hannover, Germany; Group of Primary Immunodeficiencies, Universidad de Antioquia, Medellin, Colombia; Division of Pediatric Allergy and Clinical Immunology, McGill University Health Center, Montreal, QC Canada; University of São Paulo, São Paulo, Brazil; Immunology Day Centre, Royal Group of Hospitals, Belfast, UK; Queen’s University Belfast, Belfast, UK; Child Life and Health, University of Edinburgh, Edinburgh, UK; Department of Paediatric Gastroenterology, Royal Hospital for Sick Children, Glasgow, UK; Department of Genetics, Aarhus University Hospital, Aarhus, Denmark; Royal Free Hospital, University College London, London, UK; Department of Pediatrics, University Hospitals Leuven, Leuven, Belgium; Department of Microbiology and Immunology, Experimental Laboratory Immunology, Katholieke Universiteit Leuven, Leuven, Belgium; Center for Diagnosis and Treatment of Primary Immunodeficiencies, Department of Pediatrics, Jessenius Faculty of Medicine, Comenius University in Bratislava, Martin, Slovak Republic; Department of Paediatrics, University Medical Center Hamburg-Eppendorf, Hamburg, Germany; Immuno Deficiency Center Leipzig, Clinic St. Georg, Leipzig, Germany; Translational Centre for Regenerative Medicine, University Leipzig, Leipzig, Germany; Division of Rheumatology and Immunology, Children’s Hospital Central California, Madera, CA USA; University of Washington and Seattle Children’s Research Institute, Seattle, WA USA; Department of Pediatrics and Immunology, University of Washington, Seattle, WA USA; DZIF Center, Standort Freiburg, Germany

**Keywords:** Chronic mucocutaneous candidiasis, CMC, primary immunodeficiency, PID, signal transducer and activator of transcription 1, STAT1, gain-of-function, GOF, phosphorylation

## Abstract

**Purpose:**

Gain-of-function (GOF) mutations in the signal transducer and activator of transcription 1 (*STAT1*) result in unbalanced STAT signaling and cause immune dysregulation and immunodeficiency. The latter is often characterized by the susceptibility to recurrent *Candida* infections, resulting in the clinical picture of chronic mucocutaneous candidiasis (CMC). This study aims to assess the frequency of GOF *STAT1* mutations in a large international cohort of CMC patients.

**Methods:**

*STAT1* was sequenced in genomic DNA from 57 CMC patients and 35 healthy family members. The functional relevance of nine different *STAT1* variants was shown by flow cytometric analysis of STAT1 phosphorylation in patients’ peripheral blood cells (PBMC) after stimulation with interferon (IFN)-α, IFN-γ or interleukin-27 respectively. Extended clinical data sets were collected and summarized for 26 patients.

**Results:**

Heterozygous mutations within *STAT1* were identified in 35 of 57 CMC patients (61 %). Out of 39 familial cases from 11 families, 26 patients (67 %) from 9 families and out of 18 sporadic cases, 9 patients (50 %) were shown to have heterozygous mutations within *STAT1*. Thirteen distinct *STAT1* mutations are reported in this paper. Eight of these mutations are known to cause CMC (p.M202V, p.A267V, p.R274W, p.R274Q, p.T385M, p.K388E, p.N397D, and p.F404Y). However, five *STAT1* variants (p.F172L, p.Y287D, p.P293S, p.T385K and p.S466R) have not been reported before in CMC patients.

**Conclusion:**

*STAT1* mutations are frequently observed in patients suffering from CMC. Thus, sequence analysis of *STAT1* in CMC patients is advised. Measurement of IFN- or IL-induced STAT1 phosphorylation in PBMC provides a fast and reliable diagnostic tool and should be carried out in addition to genetic testing.

## Introduction

Chronic mucocutaneous candidiasis (CMC) constitutes a collective term for a heterogeneous group of syndromes with the common feature of chronic non-invasive *Candida* infections of the skin, nails and mucous membranes, primarily with *Candida albicans*. Reasons behind the susceptibility to *Candida* infections are manifold. Infection with the human immunodeficiency virus as well as the prolonged use of glucocorticoids or antibiotics predispose to fungal infections, but the disease may also manifest as part of rare primary immunodeficiencies caused by monogenic Mendelian traits affecting the cell-mediated immunity necessary for fighting *Candida* infections [1–4]. CMC presents heterogeneously both in clinical manifestations and genetic background, however, studies conducted so far emphasize the key role of T helper 17 (Th17) cells and the impaired effector function of their cytokines interleukin 17 (IL-17) and interleukin 22 (IL-22). These cytokines have been shown to be essential for mucocutaneous anti-fungal host defense [5–7]. Indeed, patients with the autosomal dominant form of hyper IgE syndrome (HIES) have severely reduced numbers of IL-17 producing circulating T cells due to dominant-negative mutations of the signal transducer and activator of transcription 3 (*STAT3*), and often suffer from CMC [8–11]. CMC has also been reported in a large multiplex kindred segregating an autosomal recessive mutation in the caspase recruitment domain-containing protein 9 (*CARD9*). Other important infectious phenotypes reported in this family were dermatophytosis and *Candida* meningitis [12]. Patients with autoimmune polyendocrinopathy candidiasis and ectodermal dystrophy (APECED)-syndrome bearing biallelic mutations in the autoimmune regulator (*AIRE*) gene are as well susceptible to *Candida* infections but no other pathogens. These patients have high titers of neutralizing autoantibodies against IL-17A, IL-17F and IL-22 [13–15]. Patients with heterozygous *IL17F* mutations and homozygous *IL17RA* or *IL17RC* mutations have impaired IL-17 signaling and suffer from CMC [16, 17]. Furthermore, a biallelic *ACT1* deficiency has been shown to underlie one consanguineous CMC family [18]. Finally, gain-of-function (GOF) missense mutations in the signal transducer and activator of transcription 1 (*STAT1*) were shown to cause autosomal dominant familial CMC, often associated with thyroid disease, and represent the most common genetic etiology of CMC [19, 20]. The majority of GOF-*STAT1* mutations are confined to the coiled-coil domain (CCD) of *STAT1*, however, several other GOF mutations have been found in the DNA-binding domain (DBD) [19–33]. Besides increasing the susceptibility to candidiasis, several *STAT1* GOF mutations are also associated with other fungal infections such as coccidioidomycosis or histoplasmosis [34]. Analysis of mutated STAT1 proteins revealed a prolonged phosphorylation leading to prolonged transcription factor activity. Increased STAT1 responses to the interferons α/β and γ as well as IL-27 were shown to repress the differentiation of IL-17 producing T cells through mechanisms that are not yet completely understood [20, 24, 26]. The identification of genetic defects in patients with CMC offers the opportunity to confirm the diagnosis in both familial and sporadic CMC patients, to provide genetic counseling, and to enable a more precise classification of CMC. In this study, we have explored the frequency of *STAT1* mutations in a large cohort of 92 individuals consisting of 57 patients with CMC (39 familial cases and 18 sporadic cases) and 35 healthy relatives from 11 unrelated families. We analyzed patients and healthy family members in order to elucidate the underlying genetic defect and tested interferon (IFN)- and IL-stimulated STAT1 phosphorylation in patients’ peripheral blood cells by flow cytometric analysis. We compiled an in depth description of the *STAT1* clinical CMC phenotype in order to provide a clear clinical picture for this condition.

## Methods

### Patients and Controls

Inclusion criteria for this study were i) the clinical diagnosis of CMC according to the referring immunologist, ii) the availability of genomic DNA, and iii) a signed consent form for genetic research. Candidiasis was proven by swab, biopsy, or the combination of both in at least one affected family member and in every sporadic patient. The study was approved by the local ethics review boards of the University College London and the University Medical Center Freiburg. Three of the 11 families originated from Germany and three other families from Norway. One family each came from Brazil, Canada, Colombia, Slovakia and the USA. Additionally, we included six sporadic patients from the UK, three from Germany, two respectively from Colombia and Belgium and one patient each from Brazil, Canada, Denmark, India and the USA (Table 1). Due to unavailability of previous clinical data and loss-to-follow up of several patients, the clinical analysis was limited to 26 of 35 patients carrying heterozygous *STAT1* mutations.

**Table 1:** CMC cohort, *STAT1* mutations

ID	Origin	Investigated CMC patients	Mutation carriers	Invest. healthy members	Mutation carriers	Affected domain	Affected exon	BP change	AA change
**Families**									
Fam01	BRA	2	2	0	0	Coiled-coil	8	c.604A > G	p.M202V
Fam02	USA	7	4	6	0	Coiled-coil	10	c.800C > T	p.A267V
Fam03	NOR	3	3	8	0	Coiled-coil	10	c.800C > T	p.A267V
Fam04	NOR	3	3	7	0	Coiled-coil	10	c.820C > T	p.R274W
Fam05	GER	3	3	0	0	Coiled-coil	10	c.820C > T	p.R274W
Fam06	GER	4	4	3	1	Coiled-coil	10	c.821G > A	p.R274Q
Fam07	SVK	2	2	1	0	Coiled-coil	10	c.877C > T	p.P293S
Fam08	GER	2	2	0	0	DNA-binding	14	c.1189A > G	p.N397D
Fam09	CAN	3	3	0	0	DNA-binding	14	c.1211 T > A	p.F404Y
Fam10	NOR	3	0	6	0	No mutation found	n.a.	n.a.	n.a.
Fam11	COL	7	0	4	0	No mutation found	n.a.	n.a.	n.a.
Total		39	26	35	1				
**Sporadic patients**									
Spor01	GER	1	1	n.a.	n.a.	Coiled-coil	7	c.514 T > C	p.F172L
Spor02	UK	1	1	n.a.	n.a.	Coiled-coil	10	c.859 T > G	p.Y287D
Spor03	BEL	1	1	n.a.	n.a.	DNA-binding	14	c.1154C > T	p.T385M
Spor04	BRA	1	1	n.a.	n.a.	DNA-binding	14	c.1154C > T	p.T385M
Spor05	COL	1	1	n.a.	n.a.	DNA-binding	14	c.1154C > T	p.T385M
Spor06	GER	1	1	n.a.	n.a.	DNA-binding	14	c.1154C > T	p.T385M
Spor07	USA	1	1	n.a.	n.a.	DNA-binding	14	c.1154C > A	p.T385K
Spor08	BEL	1	1	n.a.	n.a.	DNA-binding	14	c.1162A > G	p.K388E
Spor09	UK	1	1	n.a.	n.a.	DNA-binding	17	c.1398C > G	p.S466R
Spor10	UK	1	0	n.a.	n.a.	(Coiled-coil)	(10)	(c.796G > A)	(p.V266I)
Spor11	UK	1	0	n.a.	n.a.	No mutation found	n.a.	n.a.	n.a.
Spor12	UK	1	0	n.a.	n.a.	No mutation found	n.a.	n.a.	n.a.
Spor13	DEN	1	0	n.a.	n.a.	No mutation found	n.a.	n.a.	n.a.
Spor14	COL	1	0	n.a.	n.a.	No mutation found	n.a.	n.a.	n.a.
Spor15	CAN	1	0	n.a.	n.a.	No mutation found	n.a.	n.a.	n.a.
Spor16	GER	1	0	n.a.	n.a.	No mutation found	n.a.	n.a.	n.a.
Spor17	IND	1	0	n.a.	n.a.	No mutation found	n.a.	n.a.	n.a.
Spor18	UK	1	0	n.a.	n.a.	No mutation found	n.a.	n.a.	n.a.
Total		18	9						

*BP* base pair, *AA* amino acid, *BEL* Belgium, *BRA* Brazil, *CAN* Canada, *COL* Colombia, *DEN* Denmark, *GER* Germany, *IND* India, *NOR* Norway, *SVK* Slovakia, *UK* United Kingdom, *USA* United States of America, *n.a*. not applicable

### PCR and Sequencing Analysis

Genomic DNA of patients and family members was isolated from whole blood (Gentra Puregene purification kit, Qiagen, Crawley, United Kingdom). To assess the presence of *STAT1* mutations, all coding exons of *STAT1* were amplified by PCR according to standard protocols with Taq polymerase (PeqLab, Fareham, United Kingdom). PCR products were purified using shrimp alkaline phosphatase (Promega, Madison, Mich) and Exonuclease I (Thermo Scientific, Waltham, Mass). Primer sequences and PCR amplification conditions are available on request. The amplified DNA fragments were subsequently sequenced with the ABI PRISM BigDye Terminator kit v3.1 (Applied Biosystems, Foster City, Calif). Sequencing was performed using the 3130xl Applied Biosystems Genetic Analyzer, DNA Sequencing Analysis software, version 5.2 (Applied Biosystems), and Sequencher, version 4.8 (Gene Codes Corp, Ann Arbor, Mich).

### STAT1 Phosphorylation

Peripheral blood mononuclear cells (PBMC) were isolated from EDTA-blood by Ficoll centrifugation. Intracellular monocyte staining: 1 × 10^6^ PBMC were stimulated for 15 min at 37 °C with IFN-α (500 U/ml; Miltenyi Biotec) or IFN-γ (100 ng/ml; Miltenyi Biotec). Intracellular CD4 staining: PBMC were first stained with anti-CD4 (Ancell; clone QS4120) and then stimulated at 37 °C with either IL-27 (200 ng/ml) or IFN-α (10,000 U/ml) for 7.5 min, 15 min and 30 min. Stimulation was stopped by adding lysis and fixation buffer (Phosflow lyse/fix; BD) followed by incubation for 10 min at 37 °C. Cells were permeabilized for 30 min on ice with pre-cooled Phosflow PermIII buffer (BD) prior to staining with anti-phospho-STAT1 (pY701) (BD; clone 4a) and anti-CD14, when monocytes were studied (Beckman Coulter; clone RMO52). Data acquisition was done using either Navios flow cytometer (Beckman Coulter) or BD LSR II (BD Biosciences). FlowJo v7.2.5/v10.0.7 (Treestar) was used for data analysis.

## Results

### Identification of STAT1 Mutations in 61 % of all CMC Patients

**Fig. 1:**
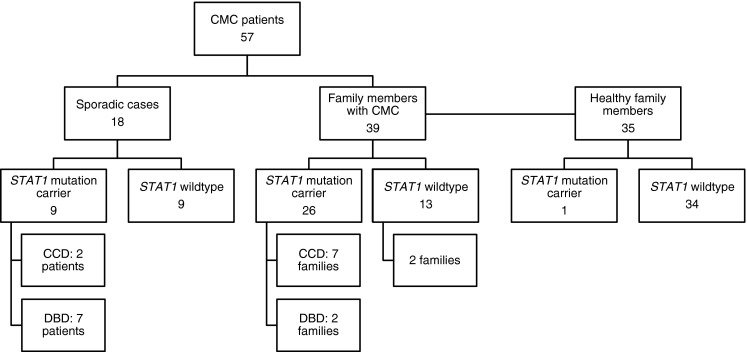
Flow chart of *STAT1* mutation detection results. The cohort consists of 92 individuals, including 18 sporadic and 39 familial patients, and 35 healthy family members

**Fig. 2:**
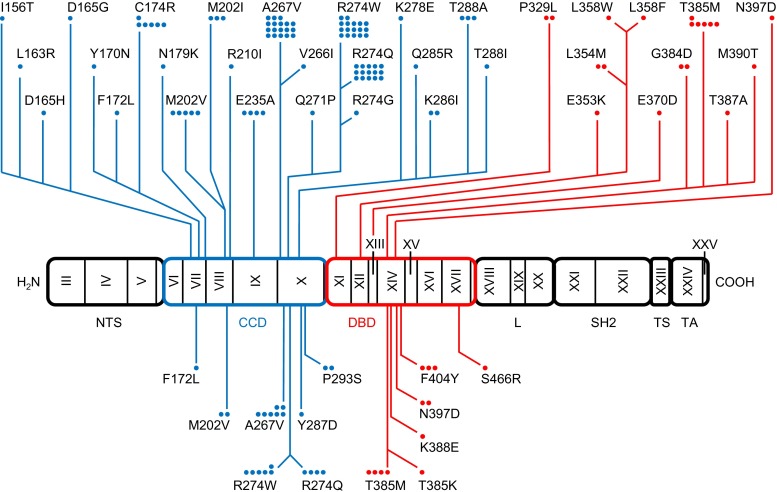
GOF *STAT1* mutations. Linear representation of the human STAT1 alpha isoform with GOF mutations associated with mycoses. Coding exons are numbered with roman numerals. Regions corresponding to the N-terminal segment (NTS), coiled-coil domain (CCD), DNA binding domain (DBD), linker domain (L), SH2 domain (SH2), tail segment domain (TS) and transactivator domain (TA) are indicated by rectangles. Mutations colored in blue affect the coiled-coil domain and colored in red affect the DNA-binding domain. GOF mutations in the upper part of the Figure have been published previously [19–34, 42–45]. GOF mutations listed in the lower part were found in the cohort under study. Each dot represents one patient

Of the 57 patients with a clinical diagnosis of CMC, a total of 35 (61 %) had heterozygous mutations in *STAT1* (Fig. 1, Table 1); 26 of these cases were familial, affecting 9 of 11 families (82 %), whereas 9 of 18 sporadic cases (50 %) had *STAT1* mutations. In one sporadic patient, we identified the variation p.V266I, which has a minor allele frequency of 0.1 % and is listed on dbSNP as “rs41473544”. We classified this variant as not associated with CMC, as its functional testing did not show STAT1 hyper-phosphorylation (see below). In contrast, 22 patients (13 familial and 9 sporadic) as well as 34 of 35 healthy family members did not show any mutation in the coding regions of *STAT1* or their flanking intronic sequences. Interestingly, one so far healthy 77-year-old female family member was identified as a carrier of the mutation p.R274Q, passing the mutation on to at least three offspring, all affected by CMC. Ten CMC patients from families Fam10 and Fam11 did not harbor a mutation in *STAT1*. Moreover, three patients from Fam02 in which the mutation p.A267V segregated in four other family members (a father and his three children) did not have a *STAT1* mutation. These three patients were subsequently classified as phenocopies. Patients without *STAT1* mutation were analyzed by next generation sequencing for *CARD9*, *IL17F*, *IL17RA*, *IL17RB*, *AIRE* and *ACT1*, but no mutation was found in these genes. Overall, we found thirteen distinct heterozygous *STAT1* mutations (Table 1, Fig. 2). Eight of these have been described previously: p.M202V, p.A267V, p.R274W, p.R274Q, p.T385M, p.K388E, p.N397D and p.F404Y [19–26, 28, 30, 31]; six patients had five distinct, novel mutations: p.F172L, p.Y287D, p.T385K, p.P293S and p.S466R, that have not been reported in CMC patients before. While we reported the mutation p.F404Y recently [25], we demonstrate the functional proof of the GOF activity of this mutation below. None of the newly identified mutations is present in the population-variation data sets 1000 Genomes (www.1000genomes.org/data) and dbSNP (www.ncbi.nlm.nih.gov/projects/SNP). Seven of these thirteen mutations were located in the coiled-coil domain of *STAT1* affecting 21 familial patients (from seven different families) and two sporadic cases. Six mutations were located in the DNA binding domain affecting five patients from two families and seven sporadic patients (Table 1, Fig. 2).

### STAT1 Hyper-Phosphorylation in PBMC of CMC Patients upon IFN-α, IFN-γ and IL-27 Stimulation

**Fig. 3:**
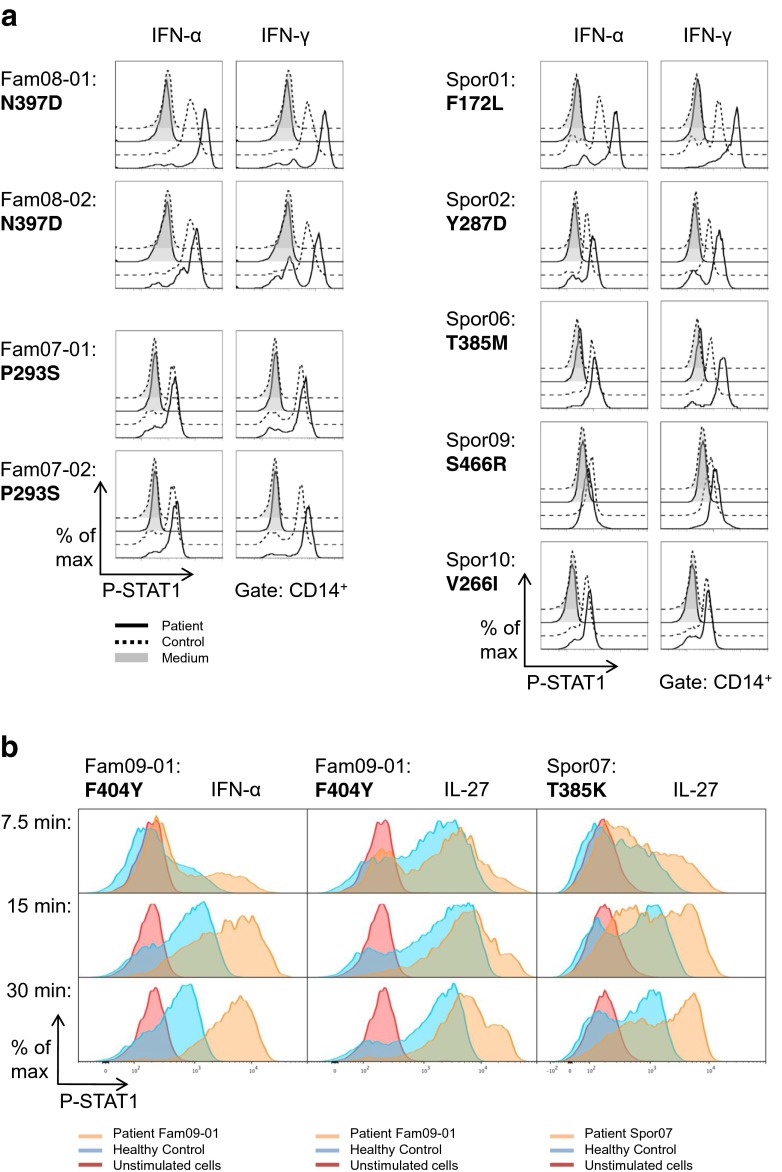
**a** Increased STAT1 phosphorylation in patients’ monocytes after IFN-α and IFN-γ treatment. PBMC from patients (solid line) and healthy controls (dashed lines) were stimulated for 15 min with IFN-α (500 U/ml) or IFN-γ (100 ng/ml). Cells were fixed and permeabilized prior to staining with anti-CD14 and anti-phospho-STAT1 (pY701) antibodies. The gate was set on CD14+ monocytes. **b** Increased STAT1 phosphorylation in patients’ CD4+ T cells following IFN-α and IL-27 stimulation. PBMC were stimulated with either IFN-α (10,000 U/ml) or IL-27 (200 ng/ml) for 7.5, 15 and 30 min. Cells were then treated as described in (**a**) and phospho-STAT1 levels were evaluated gating on CD4+ T cells (Red – unstimulated patient cells; blue – stimulated healthy control cells; orange – stimulated patient cells)

The pathogenetic outcome of CMC associated with *STAT1* mutations is assumed to be caused by hyper-phosphorylation of STAT1 [20]. In nine patients, we could assess STAT1 phosphorylation in monocytes following stimulation of PBMC with IFN-α or IFN-γ by intracellular staining with an anti-phospho-STAT1 (pY701) specific antibody and subsequent flow cytometric analysis. For the mutations p.F172L, p.Y287D, and p.N397D, STAT1 phosphorylation was clearly increased in patients’ monocytes compared to healthy controls (Fig. 3a). An increase in STAT1 phosphorylation was seen after both IFN-α and IFN-γ stimulation. However, the effect was more pronounced following IFN-γ stimulation. Monocytes from the second patient carrying the mutation p.N397D did show a moderately increased IFN-α induced STAT1 phosphorylation compared to the healthy control, whereas the mutation p.T385M in STAT1 only showed hyper-phosphorylation upon IFN-γ stimulation. These observations may point to a differential impact of certain mutations on IFN-α and IFN-γ mediated STAT1 activation. A marginal increase in STAT1 phosphorylation could be observed in monocytes with the mutations p.P293S and p.S466R, indicating that additional functional testing should be performed to prove that these mutations have an effect on STAT1 phosphorylation. No change in phosphorylation was detectable when analyzing monocytes carrying the variation p.V266I that was found to show a minor allele frequency of 0.1 %. Thus, the *STAT1* variation p.V266I is indeed unlikely to be the genetic cause for CMC in this patient, resulting in her exclusion from the 26 patients of which the clinical features are described below. In addition, we studied STAT1 phosphorylation in CD4+ T cells from four patients carrying either p.T385K or p.F404Y mutations. For this purpose PBMC were stimulated with either IL-27 or IFN-α and phospho-STAT1 levels were evaluated at different time points gating on the CD4+ population (Fig. 3b). Compared to healthy controls, patient’s CD4+ cells showed significant increase in both intensity and duration of phospho-STAT1 staining, reflected by persistent cytokine induced hyperphosphorylation at every time point tested. While this was the case for p.F404Y after IFN-α stimulation and for p.T385K after IL-27 stimulation, a relevant IL-27 induced hyper-phosphorylation of STAT1 was only apparent at later time points for the mutation p.F404Y, especially after 30 min.

### Clinical Phenotype of CMC Patients with STAT1 GOF Mutations

To determine the detailed clinical phenotype of CMC caused by STAT1 GOF mutations, we carefully reviewed data of patients previously published, and added data from our patients. For this extended clinical analysis, we were able to collect data from 26 patients (20 familial and 6 sporadic cases). The clinical phenotype for kindreds Fam02, Fam03, Fam04, Fam09 and Spor03 (Table 1) have been published previously [25, 31, 35, 36].

The main clinical feature of CMC is fungal infection of the oral mucosa. In the cohort of patients with heterozygous *STAT1* mutations, we observed episodes of oral candidiasis in 73 % (19/26) of patients, with a tendency to become chronic if untreated in 42 % (8/19) of these patients (Table 2). Antifungal treatment with e.g., azoles led to a partial remission in 62 % (10/16) of patients, 38 % (6/16) had a complete response. Moreover, 58 % (11/19) of patients needed prophylactic treatment to prevent recurrence of oral candidiasis. Aphthous stomatitis was frequent in CMC patients. A severe form was observed in 38 % (10/26) of patients, while 31 % (8/26) had moderate aphthae. Aphthous stomatitis had a lower tendency for chronicity (18 %, 3/17) compared to oral candidiasis (42 %, 8/19) and appeared with a relapsing course in the majority of affected patients (82 %, 14/17). Esophageal candidiasis was observed in two-thirds of patients (65 %, 15/23) and often showed acceptable response to treatment, with complete remission in 67 % (8/12), but relapses were frequent after treatment was stopped (87 %, 13/15). Candidiasis rarely appeared in other parts of the gastrointestinal tract. Infection of the pharynx was found in one patient (4 %, 1/23) and candidiasis of the small and large bowel was not observed.

**Table 2:** Characteristics of candidiasis

	No. of patients	% of patients
**Infection of oral mucosa**		
Oral candidiasis	19/26	73 %
Duration		
• Recurrent	11/19	58 %
• Chronic	8/19	42 %
Response to treatment		
• Complete	6/16	38 %
• Partial	10/16	62 %
• No	0/16	0 %
Aphthous stomatitis	18/26	69 %
• Severe	10/26	38 %
• Moderate	8/26	31 %
Duration		
• Recurrent	14/17	82 %
• Chronic	3/17	18 %
Response to treatment		
• Complete	6/13	46 %
• Partial	6/13	46 %
• No	1/13	8 %
**Infection of intestinal tract**		
Esophageal candidiasis	15/23	65 %
Other	1/23	4 %
Duration		
• Recurrent	13/15	87 %
• Chronic	2/15	13 %
Response to treatment		
• Complete	8/12	67 %
• Partial	4/12	33 %
• No	0/12	0 %
**Skin infections**		
Intertrigo	13/26	50 %
Pustules	12/26	46 %
Intertrigo and/or Pustules	20/26	77 %
Duration		
• Recurrent	9/18	50 %
• Chronic	9/18	50 %
Response to treatment		
• Complete	4/16	25 %
• Partial	12/16	75 %
• No	0/16	0 %
**Infection of scalp**	11/25	44 %
Duration		
• Recurrent	3/9	33 %
• Chronic	6/9	67 %
Response to treatment		
• Complete	4/8	50 %
• Partial	4/8	50 %
• No	0/8	0 %
**Infection of nails**		
Onychomycosis	16/25	64 %
Paronychia	9/23	39 %
Duration		
• Recurrent	4/17	24 %
• Chronic	13/17	76 %
Response to treatment		
• Complete	3/15	20 %
• Partial	12/15	80 %
• No	0/15	0 %
**Infection of vulva and vagina**	6/9	67 %
Limited to vulva	2/6	33 %
Extended to surrounding areas	4/6	67 %
Duration		
• Recurrent	5/6	83 %
• Chronic	1/6	17 %
Response to treatment		
• Complete	4/5	80 %
• Partial	1/5	20 %
• No	0/5	0 %

We documented a mixed picture of skin infections in the CMC cohort under study. Altogether, 50 % (13/26) of patients reported intertrigo, 46 % (12/26) pustules and 44 % (11/25) infections of the scalp; and 50 % (9/18) of affected patients reported chronic skin infections. More chronic than recurrent cases were reported, regarding infections of the scalp (chronic infections: 67 %, 6/9; recurrent infections: 33 %, 3/9). Onychomycosis appeared in 64 % (16/25) and paronychia in 39 % (7/23) of patients. Onychomycosis and paronychia were both chronic in three quarters of cases (76 %, 13/17). Treatment response varied from complete remission (20 %, 3/15) to partial remission (80 %, 12/15), rendering resistance a possible complication in patients with nail candidiasis.

Nine female patients of reproductive age provided information about vulvovaginal candidiasis. In 67 % (6/9) of these female patients *Candida* infection of the vulva was documented and candidiasis affected both vagina and perineal region in the two-thirds of cases (67 %, 4/6). Complete response after antifungal therapy was likely (80 %, 4/5), but infections of the vulvovaginal area often reoccurred (83 %, 5/6) when antifungal treatment was stopped.

The majority of patients received both topical and systemic antifungal treatment. Oral therapy with fluconazole and other triazoles was often used to provide a long-lasting prophylaxis. Oral preparations of amphotericin B were administered in severe cases. In case of topical treatment, nystatin proved to be a good alternative to triazoles. Duration of topical antifungal treatment ranged from 2 weeks to several months while medical prescription was reported to continue for about 4 weeks per episode in most cases.

Although candidiasis presented in many patients as the main or only type of infection, other infections were also observed: Dermatophytosis was reported in 14 % (3/22) of patients (Table 3). In the respective cases, *Trichophyton* species were isolated from affected skin lesions. If prevalent, bacterial infections primarily affected the respiratory tract. One or more episodes of bronchitis were observed in 41 % (9/22). Two-fifths of patients reported a history of pneumonia (41 %, 9/22). Sinusitis was also common, with 48 % (12/25) of patients giving a positive history. Furthermore, bacteria caused skin infections, mainly presenting as folliculitis (32 %, 7/22). Recurrent viral infections were rarely observed. Herpes simplex virus infections (18 %, 4/22) and papillomavirus infections (14 %, 3/22) were the most prevalent.

**Table 3:** Associated diseases

	No. of patients	% of patients
**Other Infections**		
Ears, nose and throat		
• Sinusitis	12/25	48 %
• Otitis	4/25	16 %
• Conjunctivitis	1/21	5 %
• Tonsillitis	3/25	12 %
Respiratory tract		
• Bronchitis	9/22	41 %
• Pneumonia	9/22	41 %
• Bronchiectasis	3/22	14 %
Cutaneous infections		
• Furunculosis	2/22	9 %
• Folliculitis	7/22	32 %
• Abscess	1/22	5 %
Urinary infections	1/22	5 %
Dermatophyte	3/22	14 %
Other fungi / parasites	0/22	0 %
Viral infections		
• Herpes	4/22	18 %
• Papillomavirus	3/22	14 %
• Zoster	2/22	9 %
• Severe varicella	1/22	5 %
• Epstein Barr	1/19	5 %
Sepsis	2/22	9 %
**Non-infectious diseases**		
Auto-immunity		
• Thyroid	11/25	44 %
• Alopecia	1/21	5 %
• Vitiligo	1/21	5 %
• Systemic Lupus Erythematosus	0/25	0 %
• Multiple Sclerosis	0/25	0 %
Neurological complications		
• Cerebral aneurysm	2/25	8 %
• Cerebral vasculitis	1/25	4 %
Malignancies	0/21	0 %

CMC was also associated with auto-immune conditions in our cohort. With 44 % (11/25) of patients being affected, hypothyroidism was by far the most common autoimmune condition reported. Alopecia and vitiligo were observed in one patient each (5 %, 1/21) (Table 3).

With regard to serious complications, no patient reported systemic fungal infection, and no patient had *Candida* meningitis. Other severe complications, such as cerebral aneurysm, cerebral vasculitis, and squamous cell cancer of the esophagus, have been described in other CMC cohorts [20, 37, 38]. Two unrelated patients from our cohort were reported with cerebral aneurysms. One of them developed cerebral vasculitis. Aneurysms were detected in both patients through MRI at the age of 8 and 10 years, respectively.

We further analyzed lymphocyte subpopulations, serum immunoglobulin (Ig) levels, autoantibodies, and thyroid markers in all patients from our cohort (Table 4). White blood cell counts were normal in the majority of patients (82 %, 18/22), while some patients demonstrated leukocytosis (14 %, 3/22) and one patient had slightly decreased counts (5 %, 1/22). Lymphocytes were decreased in 13 % (3/23) of patients and monocytes were elevated in 17 % (4/23). Lymphocyte subpopulations were within the normal range in the majority of patients. Counts of CD3+ T cells, CD4+ T cells, and CD19+ B cells were decreased in 10 % (2/21), 14 % (3/21) and 10 % (2/21) of patients respectively. CD3-/CD16+/CD56+ natural killer cells were decreased in 53 % (10/19) of patients. IgA was decreased in 26 % (6/23) of patients, but IgG levels, including IgG subclasses IgG1-3, were normal. Thyroid-stimulating hormone was increased in 41 % (9/22) of patients, while thyroxine and triiodothyronine were decreased in fewer patients (20 %, 4/20 and 23 %, 3/13 respectively), indicating subclinical hypothyroidism. Thyroglobulin autoantibodies were reported in 3 patients (19 %, 3/16). No thyroid carcinoma was detected in the cohort under study. Detection of other autoantibodies such as anti-microsomal, anti-insulin or anti-GAD65 antibodies occurred rarely (Table 4).

**Table 4:** Absolute lymphocyte subpopulations, serum immunoglobulins, thyroid markers and autoantibodies

	Increased		Normal		Decreased		Unknown	
	No. of patients	%	No. of patients	%	No. of patients	%	No. of patients	%
**White blood cell count**								
WBC	3/22	14 %	18/22	82 %	1/22	5 %	4/26	15 %
Granulocytes	4/23	17 %	18/23	78 %	1/23	4 %	3/26	12 %
Lymphocytes	0/23	0 %	20/23	87 %	3/23	13 %	3/26	12 %
Monocytes	4/23	17 %	19/23	83 %	0/23	0 %	3/26	12 %
Eosinophils	1/20	5 %	14/20	70 %	5/20	25 %	6/26	23 %
**Percentage of lymphocytes**								
CD3+	3/21	14 %	16/21	76 %	2/21	10 %	5/26	19 %
CD4+	0/21	0 %	18/21	86 %	3/21	14 %	5/26	19 %
CD8+	0/21	0 %	20/21	95 %	1/21	5 %	5/26	19 %
CD19+	2/21	10 %	17/21	81 %	2/21	10 %	5/26	19 %
CD16+/CD65+/CD3-	1/19	5 %	8/19	42 %	10/19	53 %	6/26	24 %
**Immunoglobulins serum levels**								
IgG	7/22	32 %	14/22	64 %	1/22	5 %	4/26	15 %
• IgG1	4/12	33 %	8/12	67 %	0/12	0 %	14/26	54 %
• IgG2	1/12	8 %	7/12	58 %	4/12	33 %	14/26	54 %
• IgG3	2/11	18 %	9/11	82 %	0/11	0 %	15/26	58 %
• IgG4	0/10	0 %	3/10	30 %	7/10	70 %	16/26	62 %
IgM	2/23	9 %	19/23	83 %	2/23	9 %	3/26	12 %
IgA	1/23	4 %	16/23	70 %	6/23	26 %	3/26	12 %
IgE	0/21	0 %	13/21	62 %	8/21	38 %	5/26	19 %
**Thyroid markers**								
TSH	9/22	41 %	12/22	55 %	1/22	5 %	4/26	15 %
T_3_	2/13	15 %	8/13	62 %	3/13	23 %	13/26	50 %
T_4_	0/20	0 %	16/20	80 %	4/20	20 %	6/26	23 %
**Autoantibodies**								
Anti-thyroglobulin antibody	3/16	19 %	13/16	81 %	n.a.		10/26	38 %
Anti-thyroid peroxidase antibody	0/21	0 %	21/21	100 %	n.a.		5/26	19 %
Anti-microsomal antibody	1/8	13 %	7/8	88 %	n.a.		18/26	69 %
Anti-adrenal antibody	0/11	0 %	11/11	100 %	n.a.		15/26	58 %
Anti-insulin antibody	1/9	11 %	8/9	89 %	n.a.		17/26	65 %
Anti-GAD_65_ antibody	1/9	11 %	8/9	89 %	n.a.		17/26	65 %
Anti-pancreatic islet cell antibody	0/9	0 %	9/9	100 %	n.a.		17/26	65 %

*n.a*. not applicable, *TSH* thyroid-stimulating hormone, *T*_*3*_ triiodothyronine, *T*_*4*_ thyroxine, *GAD*_*65*_ glutamate decarboxylase 65

## Discussion

In the present study we found that in a group of 57 CMC patients, 35 cases (61 %) had a heterozygous GOF mutation in *STAT1*. Twenty-three patients had a *STAT1* mutation in the CCD, underlining the importance of this domain as a mutation locus, while 12 had a DBD mutation in *STAT1*. The majority of families (82 %, 9/11) as well as half of the sporadic cases (50 %, 9/18) harbored a heterozygous *STAT1* mutation. Several mutations we identified in the cohort under study have been described previously (Fig. 2). Van de Veerdonk et al. published that patients of a Dutch family carrying the mutation p.R274W in the CCD had severe dermatophytosis and autoimmune phenomena (autoimmune hemolysis, pernicious anemia, autoimmune hepatitis), in addition to severe, chronic oropharyngeal candidiasis [19]. These severe autoimmune phenomena were not observed in our patients. However, the association between the *STAT1* mutation p.A267V and hypothyroidism, which has been reported [19], was also seen in two of our families.

During this study, we were able to clarify the genetic background of a large family (Fam02) with an autosomal dominant CMC type associated with hypothyroidism previously published by Atkinson et al. in 2001 [35]. The family mapped to a candidate linkage region on chromosome 2p; however, no mutation could be identified at that time. In this kindred, we detected the heterozygous *STAT1* mutation p.A267V in four of the affected family members. However, *STAT1* is located on chromosome 2q and not 2p. Only the most severely affected members with both CMC and thyroid disease harbored this *STAT1* mutation, while other family members, solely suffering from hypothyroidism, did not carry it. A likely explanation for the incorrect linkage analysis may be the misclassification of these patients due to the misleading influence of the thyroid disease, since thyroid autoimmunity is among the most common autoimmune conditions.

The high prevalence of thyroiditis which we observed in the *STAT1* mutated CMC patients in this study is an interesting aspect. One possibility for *STAT1* mutations causing hypothyroidism might be the formation of thyroid autoantibodies [20, 22]. Thyroglobulin autoantibodies (TgAb) were detected in 13 % (2/16) of patients in our cohort. TgAb are often found in patients with Hashimoto’s thyroiditis, which constitutes a common cause for hypothyroidism [39]. Furthermore, we speculate that a second mechanism may be causally related to hypothyroidism in this cohort of patients. Staab et al. assessed expression of cytokine-regulated STAT proteins in patients diagnosed as having Hashimoto’s disease or focal lymphocytic thyroiditis. In these patients, activated STAT1 dimers were detected in numerous infiltrating lymphocytes, macrophages as well as in oncocytes, while STAT3 expression was restricted to epithelial cells and showed a clear association with low levels of stromal fibrosis, suggesting that STAT3 serves as a protective factor in the remodeling of the inflamed thyroid gland [40]. The imbalance between STAT1-dependent signaling and STAT3-dependent signaling might further amplify degenerative processes in the thyroid gland. Moreover, mutated *STAT1* may contribute to hypothyroidism by interfering with thyrotropin signaling pathway. The production of suppressor of cytokine signaling 1 (SOCS1) is induced by thyrotropin, which then alters STAT1 phosphorylation [41]. Thyrotropin may act as a cytokine inhibitor in thyroid tissue in order to rescue thyroid cells. This process might be impeded by GOF *STAT1* mutations [19, 41].

We were not able to identify *STAT1* mutations in all of the CMC patients under study. Other candidate genes tested (*CARD9*, *IL17F*, *IL17RA*, *IL17RB*, *AIRE* and *ACT1*) did not reveal a molecular cause for CMC in our cohort. The cause(s) for CMC in the remainder of the cohort described here (39 %, 22/57) still needs to be identified. However, due to the high frequency of mutations, we propose that sequencing of *STAT1* should be performed in every patient suspected of having a genetic form of autosomal dominant CMC. Identification of a mutation in the CCD or the DBD in the context of clinical diagnosed CMC makes *STAT1* a likely candidate for the underlying genetic defect. Functional immunological analysis should be performed, especially when mutations are found, which have not been formally proven to result in GOF. We suggest that the analysis of the frequency of Th17 cells in peripheral blood may be performed as well as testing for IFN- and/or IL-stimulated STAT1 phosphorylation by flow cytometry. While we were able to show an increased phosphorylation of STAT1 for several mutations, however, not all *STAT1* variants seem to be disease causing, even when amino acid changes occur next to known disease causing mutations. Our functional assay indicated that e.g., p.V266I does not cause a GOF in *STAT1*. The variant V266I was previously published by Uzel et al., after being observed in an infant with an IPEX-like phenotype [28]. Uzel et al. described an enhanced DNA binding and transactivation of STAT1 for the mutation. However, this variant is also listed on dbSNP as rs41473544, the minor allele frequency is recorded to be 0.0006 in the 1000 Genomes database. When performing functional testing, we failed to observe a GOF for this variant. In summary, the relevance of this variant remains unclear. These findings emphasize the importance of complementing genetic analysis through functional investigation.

Taken together, *STAT1* is the most probable candidate gene in CMC patients but not the only gene for autosomal dominant inherited CMC. Finding the missing genetic defects still remains an interesting challenge for future research.
